# The NF-κB RelA transcription factor is not required for CD8+ T-cell function in acute viral infection and cancer

**DOI:** 10.3389/fimmu.2024.1379777

**Published:** 2024-03-05

**Authors:** Allison Voisin, Maud Plaschka, Marlène Perrin-Niquet, Julie Twardowski, Insaf Boutemine, Baptiste Eluard, Guilhem Lalle, Pierre Stéphan, Khaled Bouherrou, Laurie Tonon, Roxane Pommier, Anthony Ferrari, Ulf Klein, Mélanie Wencker, Véronique Baud, Philippe A. Cassier, Yenkel Grinberg-Bleyer

**Affiliations:** ^1^Cancer Research Center of Lyon, Labex DEV2CAN, Institut National de la Santé et de la Recherche Médicale (INSERM) 1052, Centre National de la Recherche Scientifique (CNRS) 5286, Université Claude Bernard Lyon 1, Centre Léon Bérard, Lyon, France; ^2^St. Anna Children´s Cancer Research Institute (CCRI), Vienna, Austria; ^3^Université Paris Cité, NF-κB, Différenciation et Cancer, Paris, France; ^4^Gilles Thomas Bioinformatics Platform, Fondation Synergie Lyon Cancer, Centre Léon Bérard, Lyon, France; ^5^Division of Haematology & Immunology, Leeds Institute of Medical Research at St. James’s, University of Leeds, Leeds, United Kingdom; ^6^Centre International de Recherche en Infectiologie, INSERM U1111, École Normale Supérieure de Lyon, Claude Bernard University Lyon 1, Centre National de la Recherche Scientifique (CNRS), UMR 5308, Lyon, France; ^7^Medical Oncology, Centre Léon Bérard, Lyon, France

**Keywords:** CD8 + T cells, NF-KappaB, cancer, immunotherapy, LCMV

## Abstract

CD8^+^ T cells are critical mediators of pathogen clearance and anti-tumor immunity. Although signaling pathways leading to the activation of NF-κB transcription factors have crucial functions in the regulation of immune responses, the CD8^+^ T cell-autonomous roles of the different NF-κB subunits, are still unresolved. Here, we investigated the function of the ubiquitously expressed transcription factor RelA in CD8^+^ T-cell biology using a novel mouse model and gene-edited human cells. We found that CD8^+^ T cell-specific ablation of RelA markedly altered the transcriptome of *ex vivo* stimulated cells, but maintained the proliferative capacity of both mouse and human cells. In contrast, *in vivo* experiments showed that RelA deficiency did not affect the CD8^+^ T-cell response to acute viral infection or transplanted tumors. Our data suggest that in CD8^+^ T cells, RelA is dispensable for their protective activity in pathological contexts.

## Introduction

NF-kappaB (NF-κB) is a family of transcription factors with pleiotropic functions in inflammation and immunity. The family comprises 5 subunits that share a Rel-homology domain. RelA (also known as p65, encoded by *Rela*), c-Rel and NF-κB1 are activated upon stimulation of the canonical signal transduction pathway, whereas RelB and NF-κB2 are the terminal effectors of the non-canonical (or alternative) pathway (1).

The canonical NF-κB pathway can be activated in T cells following engagement of T-Cell Receptor (TCR)/CD28, different members of the Tumor Necrosis Factor Receptor Superfamily (TNFRSF) and CD28 families, as well as several cytokine receptors, that all lead to the formation of the activating Inhibitor of KappaB Kinase (IKK)α/β/γ complex, allowing degradation of the inhibitor IκBα/β and subsequent nuclear translocation of NF-κB subunits (2). NF-κB dimers then bind to DNA to repress or activate transcription. The canonical pathway has been implicated in various aspects of T-cell biology, ranging from thymic development to effector functions (3).

Among T-cell subsets, cytotoxic CD8^+^ T cells which are critical mediators of anti-tumor and anti-pathogen immunity, have been proposed to rely on NF-κB activity at different levels, including activation, survival, proliferation, or cytokine expression (3–7). Nevertheless, the CD8^+^ T-cell-autonomous roles of NF-κB subunits, are incompletely understood. Indeed, whereas RelA is ubiquitously expressed and known as a quick and critical mediator of gene expression following stimulation, knowledge on its functions in CD8^+^ T cells is scarce. This is largely due to the embryonic lethality of mice with germline ablation of *Rela* (8). In mice, overexpression of a dominant-negative mutant form of *Rela* impaired CD8^+^ T-cell survival, as well as EOMES expression in memory T-cells (9, 10). Elegant studies have also demonstrated a direct function of RelA in IFNγ production and proliferation, in mouse and human CD8^+^ T-cells, respectively (11, 12). In patients with heterozygous, dominant negative loss-of-function (LOF) mutations in *RELA*, decreases in circulating central memory CD8^+^ T cells were reported (13, 14), although this was not confirmed in another cohort of patients (15). However, whether RelA is intrinsically required to orchestrate CD8^+^ T-cell gene expression and function *in vivo*, is unknown. Herein, we selectively ablated *Rela* in CD8^+^ T cells using a novel mouse model and engineered human cells, in order to investigate its putative roles *ex vivo* and in disease contexts.

## Materials and methods

### Mice

*Rela*-Floxed (B6.129S1-Rela^tm1Ukl/J^) mice were previously described (16). CD8^cre^ (C57BL/6-Tg(Cd8a-cre)^1Itan/J^) were a gift from Ichiro Taniuchi (RIKEN Center for Integrative Medical Sciences, Japan) (17). CD4^cre^ (Tg(CD4-cre)^1Cw1^) on a C57Bl/6 J background were purchased from the Jackson Laboratory. *Rag2*^–/–^ and C57Bl/6 CD45.1 (*Ptprc*^a^ *Pepc*^b^/BoyJ) mice were purchased from Charles River Laboratories France. Mice were bred and used in specific-pathogen-free (SPF) conditions at the CRCL animal facility (P-PAC) or the Ecole Normale Supérieure de Lyon BSL-2 facility (PBES, for LCVM experiments only). Animals were housed in individually ventilated cages with temperature-controlled conditions under a 12-h-light/dark cycle with free access to drinking water and food. Adult (6- to 30-week-old) male or female mice were used for all experiments. Studies were conducted in accordance with the animal care guidelines of the European Union and French laws. Protocols were validated by the local Animal Ethics Evaluation Committee (C2A15). Project references were #16772, 30346 and 43605.

### Human subjects

Blood samples from healthy volunteers were obtained through the Etablissement Français du Sang (EFS) (French Blood Transfusion Society).

### Tumor cell lines, transplantation and immunotherapy

MC-38 cells were a gift from Benoit Salomon (Paris, France). B16-F10 cells were purchased from the American Tissue Culture Collection (ATCC, catalog ##6475). Braf^V600E^Pten^-/-^ (Braf-Pten) cells were a gift from Julie Caramel (CRCL, Lyon, France). Cell lines were maintained in DMEM (Gibco, catalog #61965059) supplemented with 10% Fetal Bovine Serum (FBS, Thermo Fisher Scientific, catalog # 10437028) and Penicillin/Streptomycin (Gibco, catalog #15140-122). 2×10^5^ MC38 or B16-F10 or 4×10^5^ Braf-Pten cells diluted in 50 µL sterile PBS1X were injected subcutaneously into the shaved flank back of each mouse. After seven days, tumor size was monitored every two or three days with a Caliper. Tumor volume was obtained by using the formula: Width^2^ x Length. Anti-PD-L1 (Clone: 10F.9G2, catalog #BP0101) and isotype control mAbs (catalog #BP0090) were obtained from BioXCell. Mice received intraperitoneal injections of 200 µg of mAb diluted in PBS1X at D7, 9 and 11.

### Mixed bone marrow chimeras

Bone marrow cells were retrieved from tibia and femur of donor mice. Red blood cells were lysed with Ammonium-Chloride-Potassium lysis Buffer and enumerated. 1/3 WT CD45.1 bone marrow and 2/3 CD45.2 bone marrow of interest (CD4^cre^ or CD4^cre^ *Rela*^flox/flox^) were mixed to prepare bone marrow solutions. After sub-lethal irradiation (7 Gy), recipient mice were transplanted intravenously (retro orbital sinus) with 10×10^6^ bone marrow cells. Mice were given Neomycin (200 ng/mL, Sigma catalog #N6386) in drinking water for ten days. 8 weeks after reconstitution tissues were harvested for subsequent analyses.

### LCMV Armstrong infection and measurement of viral titers

Mice were infected *via* intraperitoneal injection of 2x10^5^ PFU LCMV Armstrong (a gift from Julien Marie, CRCL, France). Virus stocks were prepared using BHK21 cells, and titrated on Vero.E6 cells, following published protocols (18). Animals were weighed every other day and euthanized 10 days post infection. Spleens and livers were harvested and snap frozen in liquid nitrogen. Tissues were dissociated using ceramic beads (CK14, Ozyme catalog #OZYME003-100) and a PreCellys homogenizer, directly in lysis buffer from the Nucleospin RNA kit (Macherey-Nagel, catalog #740955.250). RNA was subsequently extracted following the manufacturer’s protocol. cDNA was synthesized using the iScript cDNA synthesis kit (BioRad, catalog #1708891). qPCR was done using a SybrGreen iTAQ kit (BioRad, catalog #1725124) on a CFX96 instrument (Biorad). The following primers were used: LCMV NP-F: CAGAAATGTTGATGCTGGACTGC; LCMV NP-R: CAGACCTTGGCTTGCTTTACACAG; LCMV GP-F: CAGACCTTGGCTTGCTTTACACAG, LCMV GP-R: GCAACTGCTGTGTTCCCGAAAC; RPLPO-F: GGACCCGAGAAGACCTCCTT; RPLPO-R: GCACATCACTCAGAATTTCAATGG (19).

### CRISPR/Cas9 gene editing in human T cells

Peripheral blood mononuclear cells were separated using Ficoll density gradient centrifugation (Eurobio, catalog #CMSMSL0101), and red blood cells were eliminated using Ammonium-Chloride-Potassium lysis buffer. Naive CD8^+^ T cells were isolated with the EasySep Human Naive CD8 T cell Isolation kit II (Stemcell, catalog #17968), following the manufacturer’s instructions. After enumeration, cells were cultured for 3 days at a concentration of 1x10^6^ cells/mL in complete RPMI 1640 W/HEPES W/GLUTAMAX-I (supplemented with 10% FBS, Penicillin/Streptomycin, Non-Essential Amino Acids, Sodium Pyruvate, and β-Mercaptoethanol, all from Gibco, catalog #72400054, 15140-122, 11140035, 11360039, 31350010, respectively), along with human IL-2 (50U/mL, Proleukin, Novartis Pharma) and Dynabeads CD3/CD28 (Thermo Fisher, catalog #11131D, at a ratio of 1 bead for 2 T cells). Ribonucleoprotein (RNP) complexes were prepared by combining crRNA (IDT, catalog #1072544), ATTO550-tracrRNA (IDT, catalog #1075928), CAS9 (TrueCut v2 cas9, Thermo Fisher, catalog # A36499), and electroporation enhancers (IDT, catalog #1075916) at equimolar concentrations in a final solution of 7.5 nmol/mL. crRNA sequences: *RELA*#1: TGCCAGAGTTTCGGTTCACT, *RELA#*2: AGCTGATGTGCACCGACAAG. The crRNA and tracrRNA were incubated at 95°C for 5 min followed by 15 min at 37°C before adding CAS9. The solutions were then incubated for 15 min at 37°C. Finally, an electroporation enhancer was included in the mix. Dynabeads were removed, cells were washed, and then suspended at 14x10^6^ cells/mL in Buffer T (Neon transfection system, Thermo Fisher, catalog #MPK10096). Cells were combined with RNP complexes so that 1.3x10^6^ cells could be electroporated in a 100 μL Neon Tip with 50 pmol of RNP. Electroporation was performed using the Neon transfection system with the following parameters: 1600V, 10 ms, 3 pulses. Subsequently, cells were transferred into 1.9 mL complete RPMI with IL-2 (20 U/mL) and Dynabeads CD3/CD28 (1 bead for 4 T cells). Cells were cultured for 3 days, washed, and stained with DAPI (Cell Signaling Technologies, catalog #4083S) in FACS buffer. DAPI^-^ ATTO550^+^ cells were sorted using a FACS ARIA II cytometer and used for subsequent assays.

### *In vitro* culture assays

#### Human T cells

Following sorting, human CD8^+^ T cells were left to rest for 2 days in complete RPMI with IL-2 (Proleukin, Novartis Pharma, 50 U/mL). Cells were then labeled using the CellTrace Violet Cell Proliferation Kit (CTV; Thermo Fisher Scientific, catalog #C34557A) and 2x10^4^ cells were stimulated with Dynabeads anti-CD3/CD28 (1 bead:4 T cells, Thermo Fisher Scientific) in complete RPMI with IL-2 (25 U/mL). After 4 days of culture, supernatants were harvested and stored at -80°C until further use and proliferation and cytokine expression were assessed by FACS.

#### Murine T cells

Naïve CD8^+^ T cells were negatively isolated using the Naïve CD8^+^ T Cell Isolation Kit (Miltenyi Biotec, catalog #130-096-543) according to the manufacturer’s instructions. Cells were labeled using the CellTrace Violet Cell Proliferation Kit (CTV; Termo Fisher Scientifc) and 5x10^4^ cells were stimulated with coated aCD3 (0.2 to 5 µg/mL, BioXCell clone 145-2C11, catalog #BE0001-1) and aCD28 (0.08 to 2 µg/mL, BioXCell clone 37.51, catalog #BE0015-1) antibodies in complete RPMI with murine IL-7 (2.5 ng/mL, Peprotech, catalog #217-17) +/- murine IL-2 (5 ng/mL, Miltenyi Biotec, catalog #130-120-332). After 4 days of culture, supernatants were harvested and stored at -80°C until further use and proliferation and cytokine expression were assessed by FACS.

### Preparation of cell suspensions

Single cell suspensions from LN, thymus and spleens were obtained by mechanical dilaceration in FACS Buffer (PBS 1X + 2% FBS, 2 mM EDTA) with glass slides, strained and washed in complete RPMI.

After being sliced in small pieces tumors were digested in RPMI 1640 (Gibco) supplemented with 1 mg/mL collagenase type IV (Sigma-Aldrich, catalog #C2674) and 250 µg/mL DNase I (Sigma-Aldrich catalog #DN25) for 25 min at 37°C followed by mechanical dissociation. Reaction was stopped by the addition of 15 mL PBS1X containing 5 mM EDTA. The solution was filtered through a 70 μm cell strainer, and any remaining solid pieces were mechanically disrupted. After centrifugation, cell pellets were resuspended in 8 mL of Percoll 40% (Sigma-Aldrich, catalog #17-08-91-01) and then carefully layered onto 4 mL of Percoll 80% in a 15 mL polypropylene tube. Tubes were centrifuged at 2,500 rpm for 20 min at RT. Mononuclear cells were retrieved from the interface of the 40:80% Percoll gradient and washed in complete RPMI.

### Flow cytometry

Cells were washed in PBS1X and incubated with purified anti-CD16/CD32 (Biolegend, catalog #101302) and a viability marker for 10 min at RT in the dark. After a wash in PBS1X cells were incubated with the surface marker antibody mix in FACS Buffer (PBS1X, 2% FBS, 2 mM EDTA) for 20 min at 4°C in the dark. Cells were then washed in FACS buffer and fixed and permeabilized using the eBioscience Foxp3/Transcription Factor Staining Buffer Set (Thermo Fisher Scientific, catalog #00-5523-00) according to the manufacturer’s instructions. Cells were washed in permeabilization buffer and incubated with the intracellular marker antibody mix for 20 min at 4°C in the dark. Cells were then washed in permeabilization buffer and resuspended in FACS buffer. At times, biotin-coupled antibodies were employed. In such instances, an additional stage of staining with fluorochrome-coupled streptavidin was required (in FACS buffer for cell surface labeling or wash buffer for intracellular labeling).

For intracellular cytokine analyses, cell suspensions were incubated 3 h with 50 ng/mL PMA (Sigma, catalog #P8139), 1 μg/mL ionoymycin (Sigma, catalog #I0634) in the presence of 1X Protein Transport Inhibitor containing Brefeldin A (BD GolgiPlug, catalog #555029) prior to staining, as mentioned above.

NP396-404 PE and GP33-41 APC class I tetramers were obtained through the NIH tetramer facility. The complete list of antibodies can be found in **Supplementary Table 1**. Acquisition was performed on a LSR Fortessa (BD Biosciences) or an Aurora spectral cytometer (Cytek Bioscience). Data were analyzed with FlowJo software v10.9.0.

### RNA-sequencing and analyses

RNA from 0.25 to 1x10^6^ CD8^+^ T cells was isolated with Nucleospin RNA extraction kits (Macherey Nagel, catalog #740955.250); libraries were prepared using an Illumina TruSeq Library Kit and sequenced with an Illumina NovaSeq instrument. Reads were aligned on reference genomes (mm10 for mouse data, GRCh38 for human data) using the STAR universal RNA-seq aligner; DEGs were calculated with DESeq2. Heatmaps were created with Morpheus (Morpheus (broadinstitute.org)). For functional enrichment analyses, we used the enricher function (default parameters) from the clusterProfiler package v4.2.2 to perform hypergeometric tests for functional enrichment analysis. Only down-regulated genes were used as the input, and the universe/background was defined as all detected genes in our RNA-Seq. The human hallmark, C2 and GOBP (C5:BP) gene sets were retrieved from the Molecular Signatures Database [MSigDB (20)] using the msigdbr function and package v7.4.1 Finally, we applied the Benjamini-Hochberg method to control false discoveries in multiple hypothesis testing.

### ELISA

ELISA were performed using “Mouse IL-2 ELISA MAX Deluxe Set”, “Mouse IFN-g ELISA MAX Deluxe Set”, “Mouse TNF-a ELISA MAX Deluxe Set”, “Human IL-2 ELISA MAX Deluxe Set”, “Human IFN-g ELISA MAX Deluxe Set”, “Human TNF-a ELISA MAX Deluxe Set” (Biolegend, catalog #431004, 430804, 430904, 431815, 430104 and 430204) and “mouse GZM-B DuoSet ELISA”, “human GZM-B DuoSet ELISA” (R&D Systems, catalog # DY1865-05 and DY2906-05) following the manufacturer’s instructions.

### Western blot

Total lysates were extracted using RIPA buffer (Invitrogen, catalog #89900) supplemented with protease and phosphatase inhibitors. Proteins were denatured for 5min at 95°C in Laemmli buffer (containing 9% SDS and 9% β-mercaptoéthanol), loaded on 10% polyacrylamide gel (Biorad, catalog #456-1035) and transferred onto PVDF membranes (Biorad, catalog #1704157) using a TransBlot Turbo apparatus (BioRad, catalog #1704150). Membranes were blocked with TBS1x-Tween 0.1% - milk 5% for 1 h at room temperature (RT) and then incubated overnight at 4°C with primary antibodies (see **Supplementary Table 1**). Membranes were washed 3 times for 10 min in TBS 1x-Tween 0.1% and incubated with corresponding HRP-coupled secondaries antibodies for 1 h at RT. Finally, membranes were washed 3 times for 10 min in TBS 1x-Tween 0.1% and detection was performed using the Immobilon Classico Western HRP substrate or the Immobilon Forte Western HRP substrate (Merck, catalog #WBLUC0500 and WBLUF0500).

### Statistics

Statistical analyses were performed using GraphPad Prism Software v9 (https://www.graphpad.com/scientific-software/prism/). For FACS data and tumor weights, two-tailed Mann-Whitney tests or paired T-tests (when 2 groups) and Kruskall-Wallis followed by Dunn’s post-test (when more than 2 groups) were used to calculate statistical significance. For tumor volume 2-way ANOVA followed by Bonferroni-Dunn’s post-test (when more than 2 groups), and two-tailed Mann-Whitney test (when only 2 groups) were used.

## Results

### RelA orchestrates mouse CD8^+^ T cell activation and gene expression at steady-state

**Figure 1:**
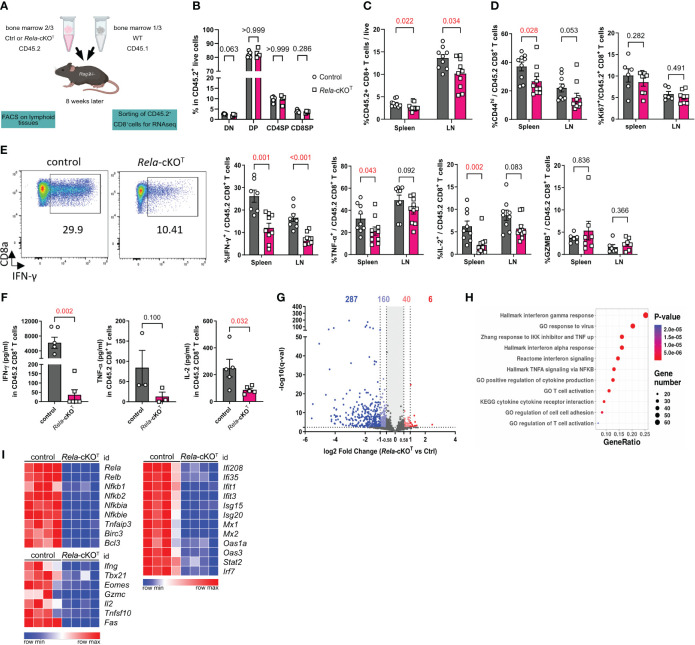
RelA shapes the transcriptome of mouse CD8^+^ T cells at steady-state. **(A)** Schematic representation of the experimental mixed bone-marrow chimera model used. **(B-E)** Thymus, spleen and LN cells were analyzed by flow cytometry. **(B)** Proportion of CD4^-^CD8^-^ (DN),CD4^+^CD8^+^ (DP), CD4^-^CD8^+^ (CD4SP), and CD4^-^CD8^+^ (CD8SP) among live CD45.2^+^ cells. **(C)** Proportion of CD45.2^+^TCRβ^+^CD4^-^CD8^+^ T cells among total live cells. **(D)** Proportion of CD44^high^ and Ki67^+^ in CD45.2^+^TCRβ^+^CD4^-^CD8^+^ T cells **(E)** Proportion of cytokines positive cells among CD45.2^+^ CD8^+^ T cells after PMA-ionomycin restimulation. **(F)** ELISA analysis of supernatants from FACS-sorted CD45.2^+^ CD8^+^ T cells after 24 h of anti-CD3/CD28 stimulation **(G-I)** RNA-seq analysis of CD45.2^+^ CD8+ T cells sorted from spleen and LN and stimulated for 4 h with anti-CD3/CD28 and IL-2. **(G)** Volcano Plot of differentially-expressed genes (DEGs). Number of DEGs up- or downregulated with a 1.5- and 2-fold change are indicated (q<0.005). **(H)** Functional enrichment analysis on down-regulated genes in *Rela*-KO CD8^+^ T cells. Representative signatures are shown. **(I)** Heatmaps of selected DEGs. FACS and ELISA data are shown as mean ± SEM of 2 independent experiments; each dot represents a mouse; Mann-Whitney tests were used. RNA-seq data are from 4 independent samples.

To investigate the T-cell autonomous functions of RelA, we used mice carrying floxed alleles of *Rela*, which we crossed with CD4^cre^ mice, resulting in the deletion of the gene across all T-cell subsets and the concomitant expression of green fluorescent protein (GFP) (16). In order to avoid indirect perturbations in CD8+ T cells that may rely on a role of RelA in other T-cell subsets, and thus specifically study the intrinsic functions of *Rela* within CD8^+^ T cells, we conducted mixed bone marrow (BM) transfer experiments of WT CD45.1^+^ cells and CD4^cre^ (control) or CD4^cre^*Rela*^F/F^ (*Rela*-cKO^T^) CD45.2^+^ cells (**Figure 1A**). Flow cytometry analyses performed 8 weeks after BM transfer, showed that the distribution of thymocyte subsets was similar regardless of the genotype (**Figure 1B**). Interestingly, analysis of secondary lymphoid organs unveiled a slight competitive disadvantage for *Rela*-cKO CD8^+^ T cells (**Figure 1C**). Among CD8^+^ T cells, a decline in CD44^high^ activated cells was detected in the absence of RelA compared to controls; however, proliferation, illustrated by Ki67 expression, remained unchanged (**Figure 1D**). As NF-κB signaling has been linked to the production of inflammatory cytokines by immune cells, we explored cytokine expression upon PMA-ionomycin restimulation *in vitro*. We observed a dramatic decrease in the proportion of IFNγ, TNFα and IL-2-expressing *Rela*-deficient CD8^+^ T cells, in the spleen and, to a lesser extent, in LN compared to controls. In contrast, Granzyme B expression was similar between groups (**Figure 1E**). These data were corroborated by measuring cytokine levels in culture supernatants of sorted CD8^+^ T cells showing decreased concentrations of IFNγ, TNFα and IL-2 (**Figure 1F**).

Seeking deeper insights into the role of RelA in regulating global gene expression, we conducted RNA-sequencing analyses on both control and mutant CD8^+^ T cells that were stimulated with anti-CD3/CD28 and IL-2 for 4 h. Differential gene expression analyses highlighted substantial changes in the transcriptome of *Rela*-cKO^T^ CD8^+^ T cells, with 293 genes significantly up- or down-regulated when applying a fold change cut-off of 2. This number increased to 493 genes when the cutoff was reduced to 1.5 (**Figure 1G**, **Supplementary Table 2**). Of note, most differentially expressed genes (DEGs) were underrepresented in *Rela*-deficient conditions, supporting a transcription-promoting function of this NF-κB subunit. Functional enrichment analysis of down-regulated genes with Gene Ontology Biological Processes (GOBP), Hallmarks and canonical pathways revealed a number of differentially enriched pathways, including NF-κB-related pathways as well as T-cell activation or response to virus (**Figure 1H**). When looking at specific gene expression, we found massive down-regulation in NF-κB pathway-related transcripts, including negative regulators (*Nfkbia*, *Tnfaip3*), but also NF-κB subunits themselves (with the exception of *Rel*), establishing the apex function of RelA in the regulation of NF-κB-driven genes. Furthermore, genes involved in T-cell function (*Ifng*, *Il2*) or maturation (*Tbx21*, *Eomes*) were down-regulated in *Rela*-deficient cells. Surprisingly, the top enriched pathways were related to IFN signaling, as the expression of many Interferon-Stimulated Genes (ISGs) was dampened in the absence of *Rela* (**Figure 1I**). Although it cannot be excluded that this phenotype stems from the reduced expression of *Ifng* itself, this confirms that, as proposed in other cell types, the NF-κB and IFN pathways are strongly interconnected.

Taken together, these observations establish RelA as a critical regulator of the CD8^+^ T-cell phenotype and transcriptome following polyclonal stimulation.

### T-cell distribution is unaffected in mice with CD8^+^ T-cell-restricted ablation of Rela

**Figure 2:**
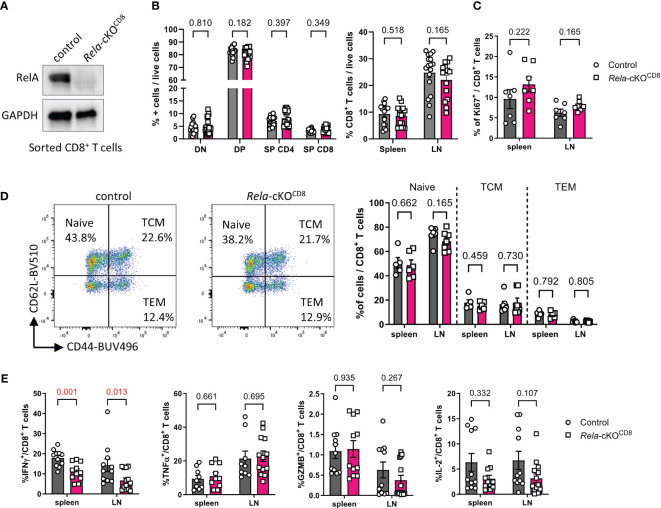
T-cell homeostasis in mice with CD8^+^ T-cell restricted ablation of *Rela.* **(A)** Western blot validation of RelA ablation in *Rela*-cKO^CD8^ CD8^+^ T cells. **(B-E)** Spleen and peripheral LN from control and *Rela*-cKO^CD8^ mice were analyzed by flow cytometry. **(B)** Proportion of live TCR-β^+^CD8^+^ cells in peripheral tissues (right) and CD4^-^CD8^-^(DN), CD4^+^CD8^+^ (DP) CD4^+^CD8^-^ (SP CD4) and CD4^-^CD8^+^ (SP CD8) in the thymus (left) among live cells. **(C)** Proportion of Ki67^+^ in CD8^+^ T cells. **(D)** Representative dot plots in the spleen and cumulative data of CD44 and CD62L expression in spleen and LN CD8^+^ T cells. TCM: T central memory; TEM: T effector memory. **(E)** Cytokine expression by CD8^+^ T cells following PMA/ionomycin restimulation, measured by FACS. Data are shown as mean ± SEM of 3-5 independent experiments; each dot represents an individual mouse; Mann-Whitney tests were used.

Next, to explore with greater specificity the CD8^+^ T-cell-autonomous functions of RelA, we crossed mice carrying *Rela*-foxed alleles with mice expressing Cre recombinase driven by a combination of the core E8I enhancer and the Cd8α promoter (called CD8^cre^ in the manuscript) (17). This allowed the conditional ablation of *Rela* and concomitant expression of GFP in peripheral CD8^+^ T cells (hereafter named *Rela*-cKO^CD8^ for conditional knock-out mice) (**Figure 2A**). Flow cytometric analyses performed on adult control and cKO animals revealed that the distribution of CD8^+^ T cells in the thymus, spleen and peripheral lymph nodes was similar between genotypes (**Figure 2B**). Moreover, levels of *in vivo* activation and proliferation, assessed by the expression of Ki67, CD44 and CD62L, were unaltered upon *Rela* ablation (**Figures 2C, D**). Following polyclonal stimulation with PMA and ionomycin, the percentage of IFNγ-producing CD8^+^ T-cells (but not TNFα, GzmB or IL-2) was reduced in *Rela*-cKO^CD8^ mice (**Figure 2E**). Hence, CD8^+^ T-cell-restricted *Rela* ablation did not strongly impact steady-state homeostasis and function of T cells, suggesting that the strong impairment in T-cell homeostasis detected in **Figure 1** likely relied on the competitive environment of mixed BM chimeras.

### Cytokine expression following *in vitro* culture of CD8^+^ T cells is altered in the absence of RelA

**Figure 3:**
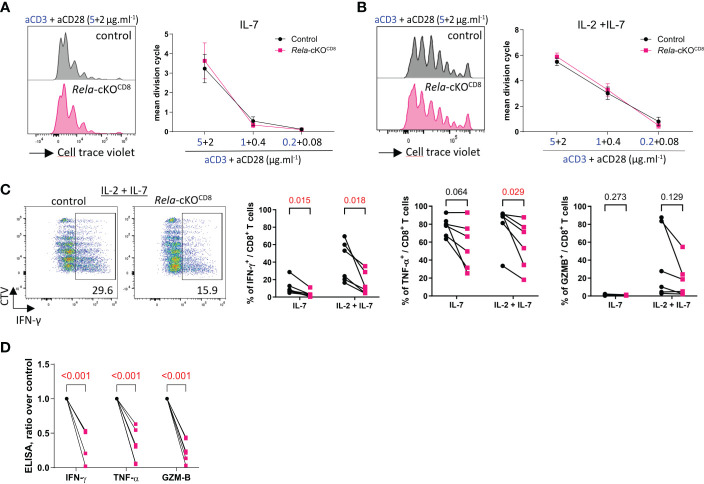
*In vitro* features of *Rela*-deficient CD8^+^ T cells. Naïve CD8^+^ T cells isolated from spleen and LN of control and *Rela*-cKO^CD8^ were stained with the CellTrace Violet Cell Proliferation Kit (CTV) and cultured with the indicated doses of anti-CD3/CD28 mAbs supplemented with IL-7 or IL-7 + IL-2 for 4 days and their phenotype was analyzed by FACS and ELISA. **(A, B)** Representative CTV profile (left) and cumulative proliferation index of live cells (right). **(C)** Representative CTV profile (left) and cytokine expression after PMA-ionomycin restimulation (right), measured by FACS. **(D)** ELISA analysis of culture supernatants. **(A, B)** Data are shown as mean ± SEM of 4 experiments; Mann-Whitney tests were used. **(C, D)** each dot represents an individual mouse from 4 independent experiments; multiple paired t-tests were used. **(D)** *Rela*-cKO samples are normalized against control samples from the same experiment.

Next, we assessed the impact of *Rela* ablation on CD8^+^ T cell responses *in vitro*. Naïve CD8^+^ T cells isolated from the spleen and LN of control and *Rela*-cKO^CD8^ mice, displayed similar levels of proliferation following 4 days of CD3/CD28 stimulation, in the presence or absence of IL-2 (**Figures 3A, B**). In contrast, we observed a dramatic impact of *Rela* ablation on the ability of CD8^+^ T cells to produce inflammatory cytokines, as illustrated by the reduction in the percentage of IFNγ- and TNFα-expressing cells as well as the quantity of secreted IFNγ, TNFα and GzmB in *Rela*-deficient cells compared to control (**Figures 3C, D**). RelA thus appears to exert critical functions in cytokine expression upon long-term TCR/CD28 engagement.

### RELA contributes to human CD8^+^ T-cell identity and functions *in vitro*

**Figure 4:**
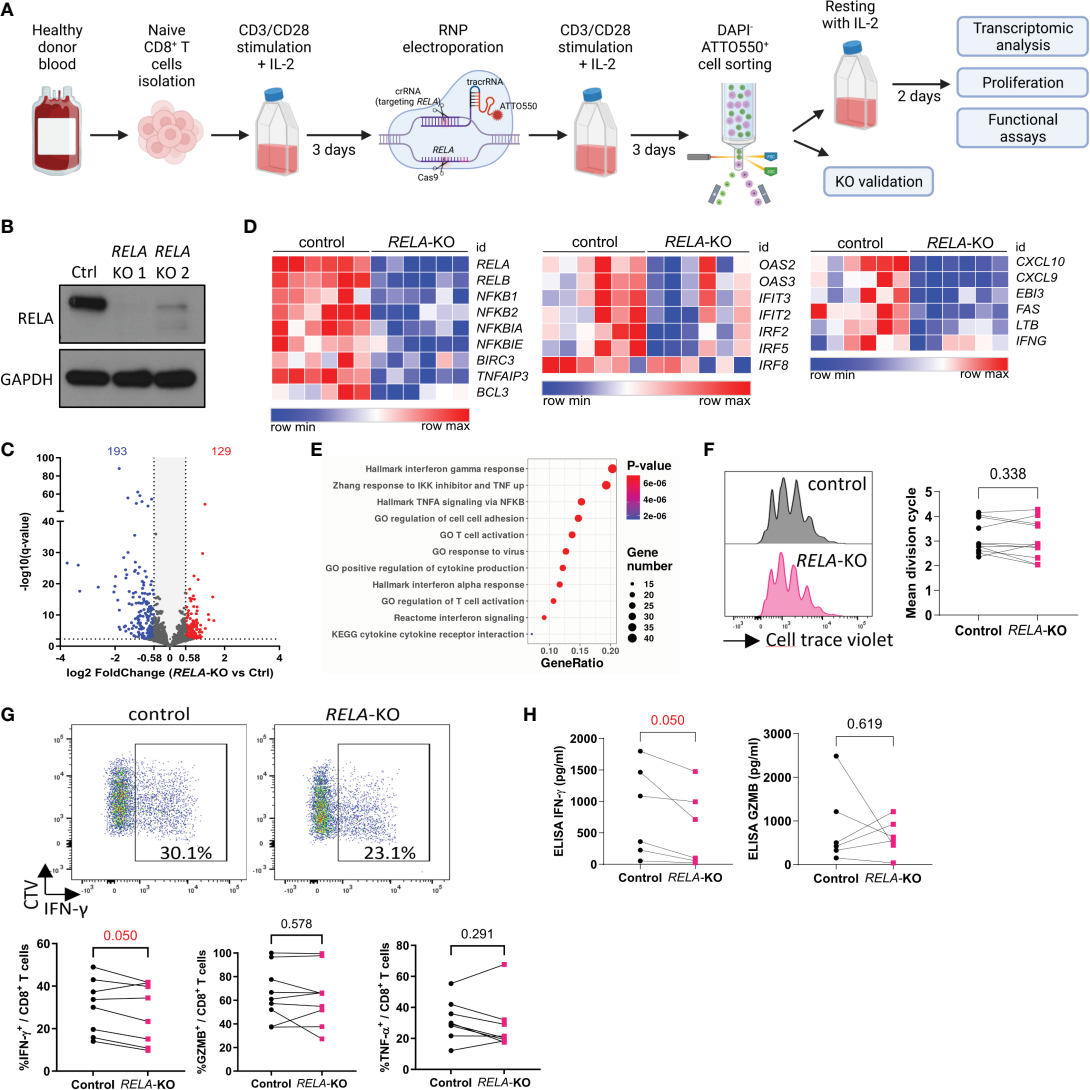
The roles of RELA in human CD8^+^ T cells. **(A)** Schematic representation of the experimental protocol. Created with BioRender.com **(B)** Western blot validation of gene editing efficacy after sorting of ATTO550^+^ live cells **(C-E)** RNA-seq analysis of 6 independent donors following 4 h stimulation with anti CD3/CD28 and IL-2. **(C)** Volcano Plot of 322 DEGs (log2 fold change >1.5, q < 0.005). **(D)** Heatmaps of selected DEGs. **(E)** Functional enrichment analysis on down-regulated genes. Representative signatures are shown. **(F-H)** *RELA*-edited CD8^+^ T cells were sorted and left to rest for two days and then labeled with CTV and stimulated 4 days with anti-CD3/CD28 and IL-2. **(F)** Representative CTV profile (left) and proliferation index of live cells from 11 donors/independent experiments. **(G, H)** Cytokines were analyzed by FACS after PMA-ionomycin restimulation **(G)** or in culture supernatant by ELISA **(H)**. In F-H, each dot represents an individual donor. Multiple paired T-tests were used. Data are from 8 donors (FACS) and 6 donors (ELISA) analyzed in independent experiments.

Although a few patients with *RELA* loss-of-function mutations have been reported, its cell-autonomous roles within human CD8^+^ T cells are unknown. We established a CRISPR-Cas9 Ribonucleoprotein electroporation protocol to ablate *RELA* in *in vitro* stimulated primary human CD8^+^ T cells from healthy donors (**Figure 4A**). RELA (encoded by *RELA*) ablation was verified by Western blotting and exceeded 80% (**Figure 4B**). We first analyzed the gene expression profiles of both normal and KO cells through RNA-sequencing after 4 h of re-stimulation with anti-CD3/CD28 and IL-2. Loss of *RELA* resulted in significant changes in the expression of 322 genes (Fold change >1.5, q-value <0.005) (**Figure 4C**, **Supplementary Table 2**). Consistent with mouse cells, enrichment analyses on down-regulated genes showed that *RELA* governed, in human CD8^+^ T cells, the expression of genes associated with the NF-κB pathway (NF-κB subunits and negative regulators of the pathway), markers of function, and cytokines, especially the response to type I and type III IFNs (**Figures 4D, E**). Thus, RELA controlled different aspects of human CD8^+^ T cell biology, suggesting a CD8^+^ T-cell-intrinsic role for *RELA* in the immunodeficiency features detected in patients with *RELA* LOF (13–15). After 4 days of stimulation with anti-CD3/28, a similar level of proliferation was observed in control and *RELA*-deficient cells, in accordance with our data from mouse experiments (**Figure 4F**). Flow cytometry and ELISA analyses showed a reduction in the expression of IFNγ-producing cells in *RELA*-deficient cells compared to control, while the expressions of TNF-α- or GZMB remained unaltered (**Figures 4G, H**).

These results reinforce the role of *RELA* in shaping the transcriptome and functions of CD8^+^ T cells both in humans and mice.

### Rela is dispensable for CD8^+^ T-cell responses during acute LCMV infection

**Figure 5:**
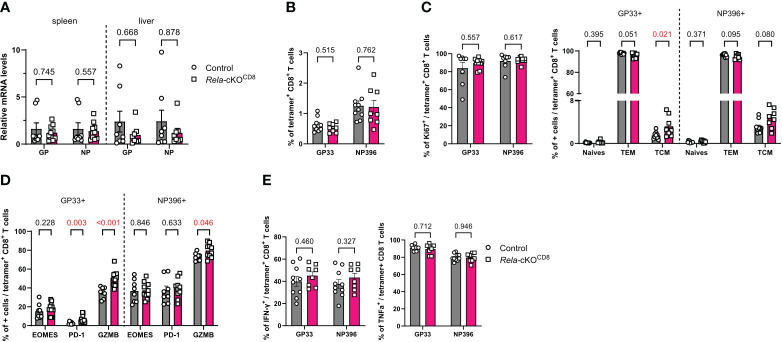
Unaltered CD8^+^ T-cell response to LCMV in the absence of RelA. CD8^cre^ (ctrl) and *Rela*-cKO^CD8^ mice were infected with 2x10^5^ PFU LCMV Armstrong **(A)** qPCR quantification of LCMV in spleen and liver 10 days after infection. **(B-E)** FACS analysis at day 10 in spleens without **(B-D)** or with **(E)** PMA-ionomycin restimulation. Each dot represents a mouse; data are shown as mean ± SEM. Mann-Whitney tests were used.

Our RNAseq data suggested altered expression of genes related to the response to viral infections in both mouse and human *Rela*-deficient CD8^+^ T-cells. To directly assess the contribution of RelA to CD8^+^ T-cell-directed antiviral responses, we infected control and *Rela*-cKO^CD8^ mice with the Armstrong strain of lymphocytic choriomeningitis virus (LCMV) that induces a strong, acute and well-defined T-cell response. Ten days post infection, the viral load, assessed by qPCR quantification of LCMV-glycoprotein (GP) and nucleoprotein (NP) encoding mRNAs, in the spleen and liver of infected mice was comparable between genotypes (**Figure 5A**). We also assessed the accumulation and phenotype of LCMV-specific CD8^+^ T cells in the spleen, using tetramers against both GP_33-41_ (GP33) and NP_396-404_ (NP396), by flow cytometry. The proportion of CD8^+^ T cells with either specificity, was unaltered in the absence of *Rela* (**Figure 5B**). Accordingly, the gross distribution of naïve/TCM/TEM compartments was similar between groups, as well as their proliferation levels (**Figure 5C**). Rather counter-intuitively, the proportion of GzmB and PD-1-expressing GP33-specific T cells was slightly increased in *Rela*-cKO^CD8^ spleens. Nevertheless, the proportion of TNFα- and IFNγ-expressing cells was not altered (**Figures 5D, E**). Altogether, these data suggest that *Rela* is dispensable for the establishment of optimal CD8^+^ T-cell responses during acute LCMV infection.

### Rela is not required for CD8^+^ T-cell anti-tumoral function and response to immune checkpoint-blockade

**Figure 6:**
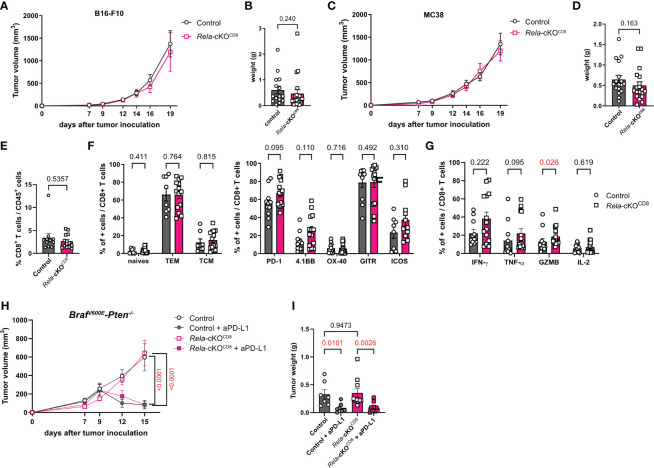
RelA in CD8^+^ T cells is dispensable for anti-tumor immunity. **(A, B)** CD8^cre^ (ctrl) and Rela-cKO^CD8^ mice were inoculated with B16-F10 melanoma cells. **(A)** Tumor volume over time (n= 16-21/group) **(B)** Tumor weight at day 19. **(C-G)** CD8^cre^ (ctrl) and Rela-cKO^CD8^ mice were inoculated with MC38 colon adenocarcinoma cells. **(C)** Tumor volume over time (n= 16-19/group) **(D)** Tumor weight at day 19. **(E, G)** FACS analysis at D19 in tumors without **(E, F)** or with **(G)** PMA-ionomycin restimulation. **(H, I)** Ctrl and Rela-cKO^CD8^ mice were transplanted with Braf^V600E^-Pten^-/-^ melanoma cells and injected with anti-PD-L1 or isotype control mAb at D7, 9 and 11 **(H)** Tumor volume over time (n= 7-10/group) **(I)** Tumor weight at day 15. Data are shown as mean ± SEM from 6 **(A, B)**, 4 **(C-G)** and 3 **(H, I)** independent experiments; each dot represents a mouse. For tumor volumes, two-tailed Mann-Whitney tests **(A, C)** or 2-way ANOVA followed by Bonferroni-Dunn’s post-tests **(H)** were used; for tumor weight and FACS data Mann-Whitney **(B, D-G)** and Kruskal-Wallis followed by uncorrected Dunn’s post-test **(I)** were used.

As CD8^+^ T cells are known to be critical actors of anti-tumor immunity, we wondered whether *Rela* was required in this context. To address this, control (CD8^cre^) and *Rela*-cKO^CD8^ mice were inoculated with B16-F10 melanoma cells. Intriguingly, *Rela* deletion had no impact on tumor growth (**Figures 6A, B**). These results were confirmed in the MC38 colon carcinoma cells (**Figures 6C, D**). Using spectral cytometry 19 days after MC38 transfer, we observed that the proportion of CD8^+^ T cells in the tumors was similar between strains (**Figure 6E**). Furthermore, the activation level of tumor-infiltrating CD8^+^ T cells was unchanged in the absence of *Rela* (**Figure 6F**), leaving the proportion of cytokine-producing cells following PMA-ionomycin restimulation unaltered, with the exception of a slight increase in GzmB^+^ CD8^+^ T cells in *Rela*-cKO^CD8^ mice (**Figure 6G**).

Given the role of CD8^+^ T cells in the response to checkpoint-blockade cancer immunotherapies, we subsequently investigated whether *Rela* activity might be a requisite for optimal response. Control (CD8^cre^) and *Rela*-cKO^CD8^ mice were transplanted with *Braf^V600E^-Pten^-/-^* melanoma cells and injected with anti-PD-L1 or isotype control mAb at D7, 9 and 11. As with the B16-F10 and MC38 models, the ablation of *Rela* did not affect tumor growth compared to control littermates treated with an isotype antibody (**Figures 6H, I**). Furthermore, PD-L1 blockade was equally efficient at inducing tumor regression in control and *Rela*-cKO mice (**Figures 6H, I**). Thus, our data indicate that *Rela* does not support antitumor immunity in CD8^+^ T cells or response to immunotherapy.

## Discussion

RelA has long been established as a master regulator of inflammation and immunity-especially through its roles in dendritic cells or macrophages. Although evidence also suggested a prominent role in shaping the CD8^+^ T-cell compartment, our study is, to the best of our knowledge, the first to directly investigate the cell-autonomous functions of RelA *in vivo.*

We show that RelA is required for the expression of numerous genes following TCR/CD28 stimulation *in vitro*, including as expected many members of the NF-κB pathway and CD8^+^ T-cell maturation drivers such as *Tbx21* or *Eomes*. In line with previous reports (11, 21), *Ifng* expression was largely down-regulated in the absence of RelA. Intriguingly, numerous IFN signaling-related genes and IFN-stimulated genes (ISGs) were also strongly impaired in mouse *Rela*-deficient CD8^+^ T cells. This critical function of RelA was further consolidated in human T cells. This link between NF-κB and IFN pathways is documented in innate immune cells (22) and our data now establish its existence in T cells. As type I and type II IFN signaling impact T-cell function (23–25), this crosstalk may have consequences on the outcome of pathological conditions. This conclusion is in stark contrast to the observations made in patients with germline *RELA* haplo-insufficiency (loss-of-function mutations), who suffer from various autoimmune or inflammatory conditions and are characterized by enhanced IFNs and ISG expression (13–15). This suggests that these phenotypes mostly relied on CD8+T-cell extrinsic functions of *RELA*.

These *in vitro* observations led us to explore whether viral infections, whose clearance largely relies on IFNs, might be impaired in *Rela*-cKO^CD8^ animals. However, *Rela* ablation did not modify LCMV loads or the priming of LCMV-specific CD8^+^ T cells. This was in line with a report showing that the absence of *Rela* in all T cells (Lck^cre^ *Rela*^F/F^ mice) led to similar responses to LCMV as those observed in control animals (26). Because complete ablation of the canonical pathway in T cells in Cd4^cre^ *Ikk2*^F/F^ mice, was shown to impair CD8^+^ T-cell cytotoxic function in cancer, resulting in enhanced tumor growth (27), we also challenged our *Rela*-cKO^CD8^ mice with different types of tumors. However, we observed that RelA was completely dispensable for CD8^+^ T-cell priming, accumulation and function in melanoma and colon adenocarcinoma. Whereas PD-1 inhibition was shown to increase NF-κB activation (28), and CD8^+^ T-cells are critical mediators of the clinical response to checkpoint-blockade cancer immunotherapies (29, 30), we also show that tumor regression induced by PD-L1 blockade is entirely maintained in *Rela*-cKO ^CD8^ animals.

Thus, at odds with our *in vitro* observations, CD8^+^ T-cell-restricted ablation of *Rela* did not drive observable phenotypes in disease contexts. This was in accordance with data from patients with *RELA* haploinsufficiency, in which no increased susceptibility to infections or cancer was reported. This could rely on different mechanisms. First, while *in vitro* T cells were stimulated with optimal levels of TCR/CD28 and IL-2/IL-7 engagement, their *in vivo* function may rely on other stimulators such as TNFSF members, which may lead to the activation of other pathways or other NF-κB subunits (31, 32). Second, it is possible that the absence of RelA *in vivo* could be compensated by other NF-κB subunits, in particular c-Rel, as shown in other contexts (33, 34). In fact, the different subunits appear to share their DNA-binding sequence and might thus be, to some extent, interchangeable *in vivo* (35–37). In this context, it would be interesting to develop mouse models that lack other NF-κB subunits in CD8^+^ T cells and even mice fully devoid of canonical NF-κB subunits, such as c-Rel and RelA-double deficient mice.

## Data availability statement

The datasets presented in this study can be found in online repositories. The names of the repository/repositories and accession number(s) can be found below: https://www.ncbi.nlm.nih.gov/geo/, GSE254685.

## Ethics statement

The studies involving humans were approved by French Blood Bank, agreement 22-093. The studies were conducted in accordance with the local legislation and institutional requirements. The human samples used in this study were acquired from a by- product of routine care or industry. Written informed consent for participation was not required from the participants or the participants’ legal guardians/next of kin in accordance with the national legislation and institutional requirements. The animal study was approved by Comité d’éthique en expérimentation animale C2A15. The study was conducted in accordance with the local legislation and institutional requirements.

## Author contributions

AV: Conceptualization, Formal Analysis, Investigation, Methodology, Visualization, Writing – original draft. MP: Data curation, Formal Analysis, Investigation, Writing – review and editing. MPN: Formal Analysis, Investigation, Validation, Writing – review and editing. JT: Formal Analysis, Investigation, Validation, Writing – review and editing. IB: Formal Analysis, Investigation, Validation, Writing – review and editing. BE: Formal Analysis, Investigation, Methodology, Writing – review and editing. GL: Formal Analysis, Investigation, Writing – review and editing. PS: Formal Analysis, Investigation, Writing – review and editing. KB: Formal Analysis, Investigation, Writing – review and editing. LT: Data curation, Formal Analysis, Writing – review and editing. RP: Data curation, Formal Analysis, Writing – review and editing. AF: Data curation, Formal Analysis, Writing – review and editing. UK: Resources, Writing – review and editing. MW: Investigation, Writing – review and editing. VB: Formal Analysis, Investigation, Methodology, Writing – review and editing. PC: Resources, Writing – review and editing. YGB: Conceptualization, Funding acquisition, Methodology, Supervision, Writing – original draft.
